# Medical Institute of Louisville

**Published:** 1845-12

**Authors:** 


MEDICAL INSTITUTE OF LOUISVILLE.

The Catalogue of the officers and students of this instiution will be published on the first of January, and will show the largest class that has yet been assembled in any Western school of medicine.

